# AnnoAgent: a language agent for single-cell automatic annotation

**DOI:** 10.1093/bib/bbaf631.036

**Published:** 2025-12-12

**Authors:** Yujie You, Le Zhang

**Affiliations:** School of Computer Science and Engineering, Sichuan University of Science and Engineering, Yibin, 644000, China; College of computer science, Sichuan University, Chengdu, 610065, China

## Abstract

**Backround:**

Cell annotation is a key step for characterizing cellular heterogeneity using scRNA-seq data. The accuracy of cell annotation directly affects the reliability of downstream studies. However, annotating cells by manually tools is cumbersome and time-consuming, which is hard to meet the requests for scRNA-seq data analysis.

**Methods:**

To address this issue, we introduce an LLM-driven agent framework AnnoAgent by integrating mainstream cell annotation models, which can achieve high-performance automatic annotation of cell types by playing strategist and assessor roles. Firstly, strategist will receive reference dataset and automatically performs data processing to generate training and validation matrices. Secondly, strategist employs training and validation matrices to train four annotation models, scBiGNN, CellTypist [1], SVM and scVI [2] as the implementer. Thirdly, strategist invokes implementer to predict the cell types in query dataset, and then generates metrics for each implementer. Finally, the assessor evaluates results of the comprehensive metrics and provide the final predicted cell types.

**Results:**

The evaluation results on the lung adenocarcinoma scRNA-seq dataset [3] indicate that AnnoAgent can integrate multiple mainstream cell annotation models to achieve optimal annotation performance, which not only greatly reduces the workload of scientific data analysis but also offers a new solution for precise automated annotation of scRNA-seq data.

**References:**

1. Domínguez, C. ‘Cross-tissue immune cell analysis reveals tissue-specific features in humans.’ Science, 2022;376(6594):p.eabl5197.

2. Virshup, I. ‘The scverse project provides a computational ecosystem for single-cell omics data analysis.’ Nature Biotechnology, 2023;41(5):p.604–606.

3. Huang, H. ‘S100a4+ alveolar macrophages accelerate the progression of precancerous atypical adenomatous hyperplasia by promoting the angiogenic function regulated by fatty acid metabolism.’ eLife, 2025;13:p. RP101731.

